# Retinal artery occlusion following CoronaVac injection in a 45-year-old Filipino

**DOI:** 10.3205/oc000220

**Published:** 2023-07-12

**Authors:** Daniel Jose M. Mendoza, David Francis F. Chan, Ellen N. Yu-Keh, Bryan Christopher W. Sy

**Affiliations:** 1 Department of Ophthalmology, Veterans Memorial Medical Center, Quezon City, Philippines; 2 Eye Institute, St. Lukes Medical Center, Quezon City, Philippines

**Keywords:** COVID-19 vaccine, CoronaVac, SARS-CoV-2, retinal vasoocclusion, retinal arterial occlusion, adverse effects

## Abstract

**Background:**

While complex public health challenges and the emergence of variants have impeded responses to the COVID pandemic, vaccines continue to represent a crucial tool in mitigating the risk of morbidity and mortality. Safety issues weigh heavily upon both the utility and acceptability of every vaccine. Reports of sight-threatening events are scarce.

**Case description:**

We report the case of a hypertensive 45-year-old Filipino who noted unilateral (right eye) blurring of vision within 48 hours of his first dose of CoronaVac (Sinovac, China), an inactivated SARS-CoV-2/COVID-19 vaccine, with macular retinal arterial occlusion noted on day 21 post-inoculation. Further work-up revealed abnormal glycemic, metabolic, inflammatory, and bleeding parameters. Vision improved from counting fingers to 20/100 at week 6 with no interventions.

**Conclusion:**

A potential association between retinal vasoocclusion and inoculation with CoronaVac in our patient is supported by the temporal sequence of events, multiple mechanisms put forward in other cases, and reports of vascular adverse reactions in large country-level trials. It is mitigated by the profound infrequency of such events and the potentially substantial risk for ocular ischemic events imparted by the patient’s baseline clinical background. Continued understanding of vaccine adverse reactions, however rare, is important not only for individual patient safety. This is helpful in ensuring the utility of current vaccines and in preserving the acceptability of vaccines through and beyond the current pandemic.

## Introduction

The massive rollout of COVID-19 vaccinations occurred at roughly the second year into the pandemic, with global case numbers rising above 200 million and deaths numbering more than 4.5 million. While emerging variants have continually hampered infection control and societal reopening measures, vaccines have contributed substantially to the mitigation of morbidity and mortality. Swift development resulted in multiple vaccines, with over 6 billion individual inoculations carried out at that point [1].

Vaccine safety has been highly relevant to the global public, as demonstrated by the reactions of the general public and policy adjustments following reports of vaccine-induced immune thrombotic thrombocytopenia [2]. Approved inactivated COVID vaccines meanwhile constituted a meaningful proportion of primary series vaccinations in numerous populations, including developing/mid-lower income states like the Philippines [3], [4], [5]. These types of vaccines have been used for many decades and were viewed as more predictable. Ocular adverse effects have been reported only sporadically, and sight-threatening events have been rare [5], [6], [7].

We report findings from a 45-year-old Filipino who experienced vision loss within 48 hours of his first CoronaVac (Sinovac Biotech, China) dose, examined in the fourth week following symptom onset. Decreased vision was attributable to retinal arterial occlusion, an entity bearing risk not only for lasting visual loss but acute ischemic events elsewhere, as well as death [8], [9].

## Case description

Our patient received his first dose in August 2021, experiencing undocumented fever and chills on day 1 post-vaccination, spontaneously resolving the following day. Rapid, painless diffuse vision loss followed. A nasopharyngeal reverse transcription – polymerase chain reaction (RT-PCR) swab was done on day 7 post-vaccination which returned negative. 

Persistence prompted consult at day 21 post-vaccination. Vision in the right eye was counting fingers 2 feet; in the left eye uncorrected vision was 20/20. In-clinic review of systems and physical examination were unremarkable. Blood pressure was 140/90. In the right eye grade 1 relative afferent pupillary defect was noted. Intraocular pressures of 10 mmHg, clear lenses, absence of cells and flare in both anterior and posterior segments were noted. 

Previous vision was recalled as subjectively equivalent in both eyes, with no history of ophthalmic procedures. He reported a previous diagnosis of hypertension within the past 4 years, a 32 pack-year smoking history, the absence of blood pressure records, medication use, and a history suggestive of atopy. 

**Figure 1:**
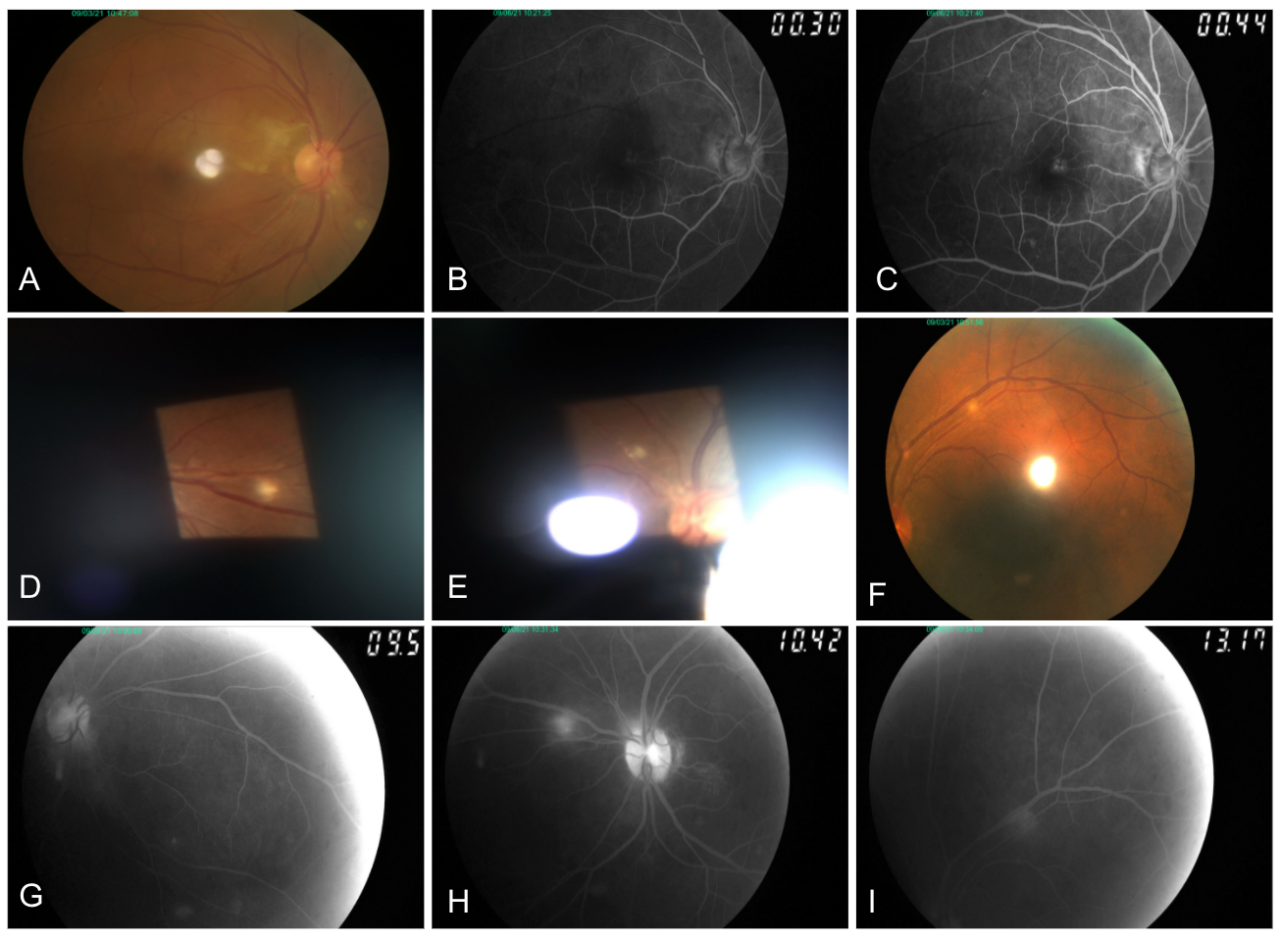
Fundus photography and fluorescein angiography of the patient’s affected right eye at the week 4 after inoculation. Gross appearance on the photographs was similar to findings in the third week after inoculation. A: Fundus photo of the right eye showing whitening of an arterial branch close to the disc (branch at <500 µm from the 11–12 o’clock disc margin) that projects temporally, passing the mid-superior papillomacular area, onto the superior para-perifovea. Flanking dot hemorrhages and background retinal whitening are seen in both areas. B–C: Delayed perfusion and superonasal para-perifoveal staining. D–F: Dull-white patches seen scattered across the retina of both eyes. G–H: Staining of the dull-white patches seen on recirculation of both eyes.

Noted on fundoscopy was the whitening of an arterial branch close to the disc (branch at <500 µm from the 11–12 o’clock disc margin) that projects temporally, passing the mid-superior papillomacular area, onto the superior para-perifovea. Flanking dot hemorrhages and background retinal whitening were seen in both areas (Figure 1A (Fig. 1)). Few (<10) dull-white patches sized ~125–500 µm were scattered in the superior fundus bilaterally (Figure 1D–F (Fig. 1)).

**Figure 2:**
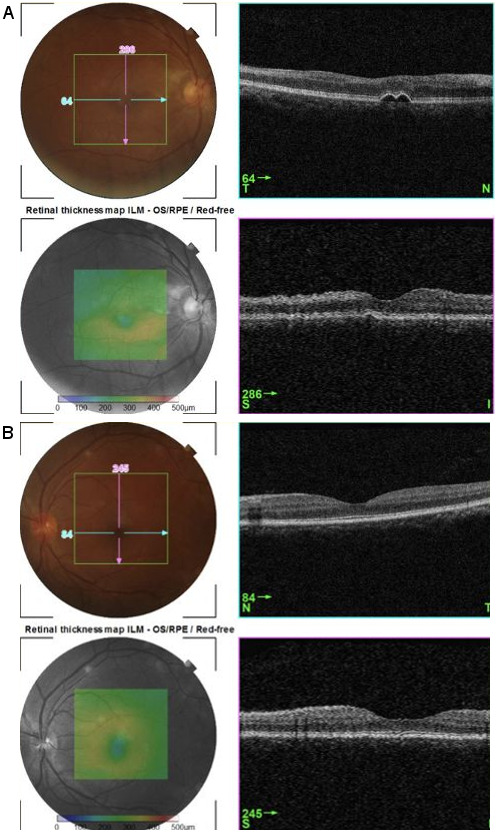
Optical coherence tomography (OCT) at week 4 after inoculation. A: Right eye OCT of the area of superonasal para-perifoveal staining seen in Figure 1C. RPE detachments are seen. B: Left eye OCT of the area of granular staining in the mid-inferior papillomacular area. Bumpy RPE-BM contour is seen.

**Figure 3:**
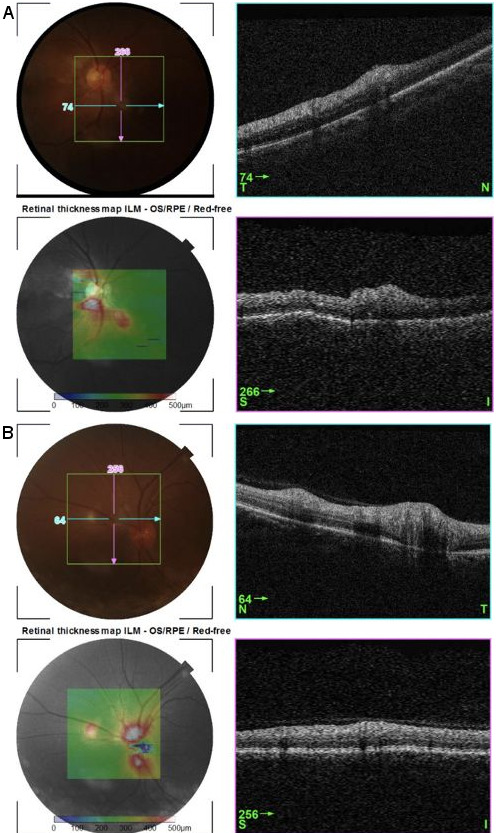
Optical coherence tomography (OCT) at week 4 after inoculation. A–B: OCT of the dull-white lesions seen in both eyes. They are rNFL/inner retinal nodularities with homogeneous mid-high reflectivity and posterior shadowing.

Fluorescein angiography revealed delayed perfusion in the described right macular areas (Figure 1A–C (Fig. 1)). Optical coherence tomography elucidated numerous findings. Noted in the right eye: (1) superior foveal thinning with intact central foveal depression, and (2) focal retinal pigment epithelium (RPE) detachments in the area of granular staining in the superonasal para-perifovea (Figure 2A (Fig. 2), which corresponds to Figure 1C (Fig. 1)). Noted in the left eye: bumpy RPE-basement membrane contour in an area of staining in the mid-inferior papillomacular area (Figure 2B (Fig. 2)). The dull-white lesions demonstrated well-defined staining and were seen as retinal nerve fiber layer (rNFL) – level nodularities with homogeneous mid-high reflectivity and some posterior shadowing (Figure 3A–B (Fig. 3)).

Work-up on days 21–28 post-vaccination revealed:

- Right eye perimetry: paracentral scotoma corresponding to the ischemic area
- Elevated fasting blood sugar, HbA1c, triglycerides, partial thromboplastin time, erythrocyte sedimentation rate, and eosinophil counts
- Negative D-dimer, normal/unremarkable C-reactive protein, and other complete blood/platelet count components

Vision improved to 20/100 with stable ocular findings. 

## Discussion and conclusions

Our patient presented with acute monocular retinal arterial occlusion symptomatic by 2–3 days after his first CoronaVac dose against a complex clinical background. Multiple intraocular findings were noted bilaterally – focal RPE changes, and scattered nodular inner layer lesions. Chronicity is more applicable to the former, and while the latter may be infarct-related, it can alternatively be a separate, pre-existing posterior segment pathology. Their contribution to the acute ocular infarct can neither be eliminated nor firmly related definitively. 

Global risk for ischemic events meanwhile was likely present beforehand. Despite the absence of retinal changes suggestive of chronic hypertension or hyperglycemia, cardiovascular and metabolic perturbations were likely present prior to inoculation per work-up done. The occurrence of a pervasive inflammatory condition was, on the other hand, not strongly supported by further work-up. Eosinophil elevation was not profound and symptoms of severe inflammatory pulmonary disease were absent – findings to be expected from vaccine-related Th2-mediated reactions [10].

No reports exist of widespread acute ischemic events among the many hypertensives, diabetics, or patients with chronic immunologic or rheumatologic conditions immunized with inactivated COVID vaccines. Reports of ocular/vision-related adverse events are extremely scarce – none were reported in two early national efficacy/effec-tiveness trials, while as of 26 June 2022, 292 cases of various ophthalmic adverse effects were recorded locally (consolidated with BBIBP-CorV/Sinopharm, from 46,802,122 total doses administered) [5], [6], [7]. Interestingly, a study of optical coherence tomography angiography (OCT-A) measurements of foveal and optic disc vascular densities within one week of CoronaVac first dose administration found no significant change in values among forty healthcare professionals with no systemic comorbidities [11]. Thus, should an association with vaccination be present in our case, the hypothesis we favor is that his unique combination of baseline characteristics and/or (an) undocumented condition(s) may have imparted a uniquely low tolerance of the varied immunologic events following inoculation.

One or a combination of the numerous mechanisms explored in the literature may thus have been at play; the diversity of presentations appears to suggest complex processes are involved [12]. For CoronaVac, autoimmune/inflammatory syndrome induced by adjuvants (ASIA) has been examined in cases of thyroiditis, and acute arterial vasospasm was the postulated mechanism in a case of acute (40 minutes post-second dose) self-resolving left congruous hemianopia [13], [14]. Notably, a case series covering the same time period reported two Brazilian females manifesting also with arterial occlusions following CoronaVac inoculation. Unlike our patient, both noted visual symptoms later (7 and 15 days post-injection) and after second doses, had no systemic risk factors, eventually yielded signs and symptoms suggestive of Susac Syndrome, and had previous COVID-19 infection. Such uncommon immunologic pathologies – including history of COVID-19 infection – may serve to increase risk for an acute microvascular event such as retinal occlusion [15]. Further, should our patient indeed have had a pre-existing ocular comorbidity such as a chorioretinopathy or uveitis (white dot syndrome), a pathologic antigen-mediated response unique to the eye is a theoretical possibility, however improbable [12], [16].

Mechanisms that may occur with other vaccines and vaccine types cannot be considered totally exclusive as well. For the other inactivated vaccine, BBIBP-CorV/Sinopharm, ocular inflammation following inoculation in seven cases has been related with numerous mechanisms including molecular mimicry [17]. Antibody-dependent enhancement is more closely associated with inactivated vaccines in general, although based on early trials expectations for the vaccines in question are reduced [18]. With regards mRNA and adenoviral-vectored COVID-19 vaccines, varied entities of retinal infarcts were noted in a series of 6 patients with different ages and different comorbidity profiles at 2–12 days following different dose numbers. A multitude of risk factors were cited; of note, the 2 cases with arterial occlusion were somewhat diverse. Though having controlled or non-treatment requiring comorbidities, they were of middle and advanced age, and presented at 3 and 12 days post second dose. Febrile/flu-like symptoms as seen in our patient were cited as a possible precedent for hypovolemia and subsequent ischemia in a case of bilateral acute macular neuroretinopathy. In all, events leading to thromboembolic phenomena may be the common pathway involved [19]. 

For our patient, while deficiencies in work-up may be significant – absence of brain imaging, ultrasonography for ocular and/or carotid blood flow, cardiac studies (electro- and echocardiography), and a more extensive coagulation profile (such as Factor V Leiden, etc.) – no signals we documented appear to favor a particular mechanism. We, however, continue to perceive a potential relationship with vaccination due to the relatively immediate onset of symptoms following first dosing against the backdrop of numerous metabolic derangements documented weeks later. 

As is stated in other case reports and series, firmer relationships may be established with bigger numbers of patients/controls and more extensive work-ups. This report contributes to this effort, submitting that while retinal occlusions are profoundly rare if related to vaccination, continuous vigilance for such occurrences is important. Identification of susceptible individuals may prevent disability and death, help preserve the acceptability of approved vaccines, and ultimately avert more destructive consequences within and across societies.

## Abbreviations

- RT-PCR: Reverse transcription – polymerase chain reaction
- RPE: Retinal pigment epithelium
- rNFL: Retinal nerve fiber layer
- ASIA: Autoimmune/inflammatory syndrome induced by adjuvants

## Notes

### Patient consent

Written informed consent was obtained from the patient for the publication of this case report and accompanying images. 

### Ethics statement

No deviations from standards of care occurred, and no experimental measures were undertaken necessitating ethics approval. 

Outputs from diagnostics and clinical data are secure and may be made available where lawful and with consent. The datasets used and/or analyzed during the current study are available from the corresponding author on reasonable request.

### Authors’ contributions

All authors discussed and approved the diagnostic and management interventions for the patient. DJMM and DFFC created the first drafts of the manuscript. All authors finalized and approved the submitted version.

### Acknowledgements

We thank the Novagen Eye Center and Providence Hospital Eye Center for facilitating numerous diagnostics and consultation sessions with the patient.

### Competing interests

The authors declare that they have no competing interests.
